# Macular Burns from Nonmedical Lasers

**DOI:** 10.4274/tjo.29577

**Published:** 2016-06-06

**Authors:** Işıl Sayman Muslubaş, Mümin Hocaoğlu, Serra Arf, Hakan Özdemir, Murat Karaçorlu

**Affiliations:** 1 İstanbul Retina Institute, İstanbul, Turkey

**Keywords:** Nonmedical laser, macular burn, vitreoretinal surgery

## Abstract

Laser devices are widely used for medical, military, industrial and entertainment purposes. This extensive and unregulated use of lasers can cause a variety of maculopathies that can result in permanent vision loss. Uncontrolled and inappropriate use of laser instruments should be prevented with strict rules. We strongly emphasize the importance of changing the general misperception that lasers are safe to use for entertainment purposes. In this study we aim to report the clinical features of three patients with a history of maculopathy caused by exposure to laser light in an entertainment venue.

## INTRODUCTION

The laser, whose name originated as an acronym for ‘Light Amplification by Stimulated Emission of Radiation’, was first developed in 1960.^1^ Following animal studies, laser devices became widely used in a range of fields for medical, industrial, research and entertainment purposes. As the usage of lasers became more common, the incidence of laser-related injuries also increased.^2^ Because visible and near-infrared light are focused and concentrated on the retina, this tissue is most susceptible to laser-related injury.^3^ Reports of retinal laser injuries include subretinal hemorrhage, retinal edema, damage to the retinal pigment epithelium, vitreous or chorioretinal hemorrhage, perifoveal pigment changes or deposits, foveal ring-shaped hypopigmented lesions and rarely, choroidal neovascularization.^4^ We aimed to present two cases of foveal subhyaloid hemorrhage and one case of subfoveal intraretinal hemorrhage associated with laser burns.

## CASE REPORTS

### Case 1

**Figure 1:**
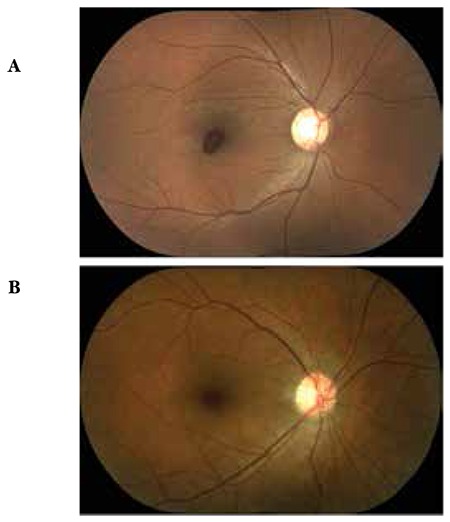
Case 1, preoperative (A) and postoperative (B) color fundus photographs

**Figure 2:**
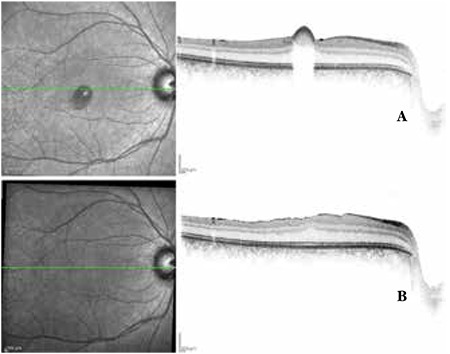
Case 1, preoperative (A) and postoperative (B) optical coherence tomography images

**Figure 3:**
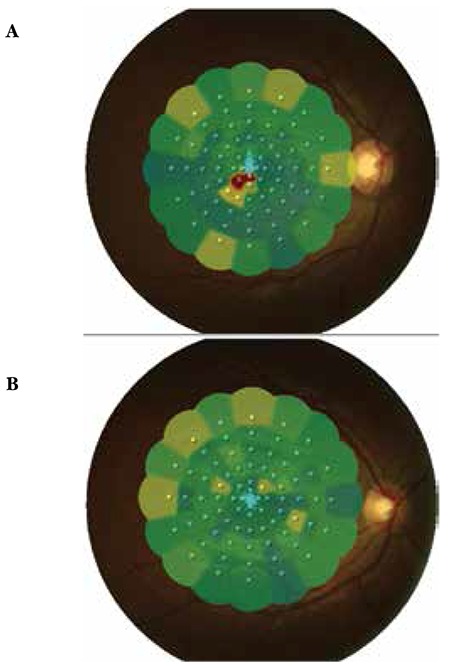
Case 1, preoperative (A) and postoperative (B) microperimetry-1 images

A 28-year-old male presented to our clinic complaining of loss of central vision in the right eye. He had attended a wedding three days earlier where his right eye was exposed to laser light. On ophthalmologic examination, his best corrected visual acuity (BCVA) was 3/10 in the right eye and 10/10 in the left eye. Anterior segment examination was normal and intraocular pressure (IOP) was 15 mmHg in both eyes. On fundus examination, foveal hemorrhage was observed in the right eye, while the left eye was normal (Figure 1A). On optical coherence tomography (OCT) performed on the same day, the foveal cross-section of the right eye revealed a hyperreflective dome-shaped protrusion extending into the vitreous cavity due to hemorrhage under the internal limiting membrane (ILM). This protrusion caused optical shadowing of the underlying tissue layers (Figure 2A). The right eye appeared normal. On microperimetry-1 (MP1) analysis, fixation was predominantly eccentric and stable and retinal sensitivity in the central 20 degrees was measured as 16.7 decibels (dB) in the right eye. Absolute and relative scotoma was present centrally in the area of hemorrhage (Figure 3A). Fixation was central and stable in the left eye, and retinal sensitivity in the central 20 degree field was normal.

Surgery was planned for the right eye after informing the patient about his current condition, natural disease course, and the risks and success rates associated with the procedure. Two days later, 23-gauge (G) pars plana vitrectomy (PPV), posterior hyaloid dissection and ILM peeling were performed. The patient developed no complications in the early postoperative period and was discharged with 0.1% dexamethasone eye drops 4 times a day for 1 month, 0.3% tobramycin drops 4 times a day for 1 month and ciprofloxacin 750 mg tablet twice a day for 1 week.

Postoperative follow-up examinations were done at 1 day, 1 week, 1 month, 6 months and 1 year after the procedure. At 1-year follow-up, BCVA was 10/10 in both eyes. Anterior segment examination was normal and IOP was 18 mmHg in both eyes. Retinal attachment was observed in the right eye on fundus examination. Hemorrhage or serous elevation were not detected (Figure 1B). The left eye appeared normal. On OCT performed on the same day, foveal sections from the right eye revealed that foveal cupping and the thickness was 339 microns. In the foveal region, the photoreceptor inner segment/outer segment (IS/OS) junction and external limiting membrane (ELM) were intact (Figure 2B). OCT findings in the left eye were normal. On MP1, fixation was central and stable and retinal sensitivity was measured as 17.0 dB (Figure 3B).

### Case 2

**Figure 4:**
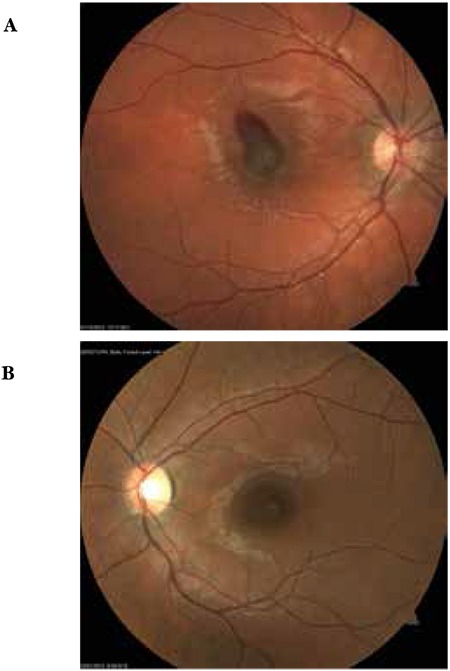
Case 2, preoperative (A) and postoperative (B) color fundus photographs

**Figure 5:**
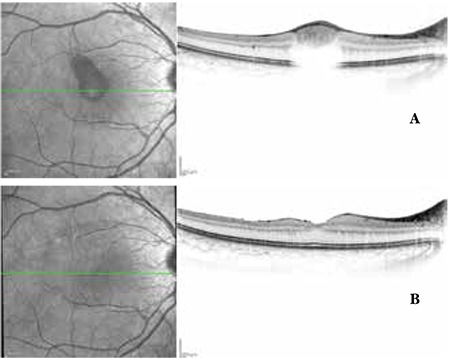
Case 2, preoperative (A) and postoperative (B) optical coherence tomography images

A 9-year-old girl was examined for complaints of blurred vision in her right eye for 15 days. According to her history, her vision problem began after looking into a laser at a hotel 15 days earlier. On ophthalmologic examination, her BCVA was counting fingers from 3 meters in the right eye and 10/10 in the left eye. Anterior segment examination was normal and IOP was 14 mmHg in both eyes. On fundus examination, foveal hemorrhage was observed in the right eye, while the left eye was normal (Figure 4A). On OCT performed on the same day, the foveal cross-section of the right eye revealed a hyperreflective dome-shaped protrusion extending into the vitreous cavity due to hemorrhage under the ILM. The foveal pit was absent (Figure 5A). The left eye appeared normal. The MP test could not be performed due to patient noncompliance.

Surgery was planned for the right eye after informing the patient about her current condition, natural disease course, and the risks and success rates associated with the procedure. Two days later, 23-G PPV, posterior hyaloid dissection and ILM peeling were performed. The patient developed no complications in the early postoperative period and was discharged with 0.1% dexamethasone eye drops 4 times a day for 1 month, 0.3% tobramycin drops 4 times a day for 1 month.

Postoperative follow-up examinations were done at 1 day, 1 week, 1 month and 6 months after the procedure. At 6 months her BCVA was 9/10 in the right eye and 10/10 in the left eye. Anterior segment examination was normal and IOP was 15 mmHg in both eyes. Retinal attachment was observed in the right eye on fundus examination. Hemorrhage or serous elevation were not detected (Figure 4B). The left eye appeared normal. On OCT performed on the same day, foveal thickness was 272 microns in the right eye. In the foveal region, the photoreceptor IS/OS junction and ELM were intact (Figure 5B). OCT findings in the left eye were normal.

### Case 3

**Figure 6:**
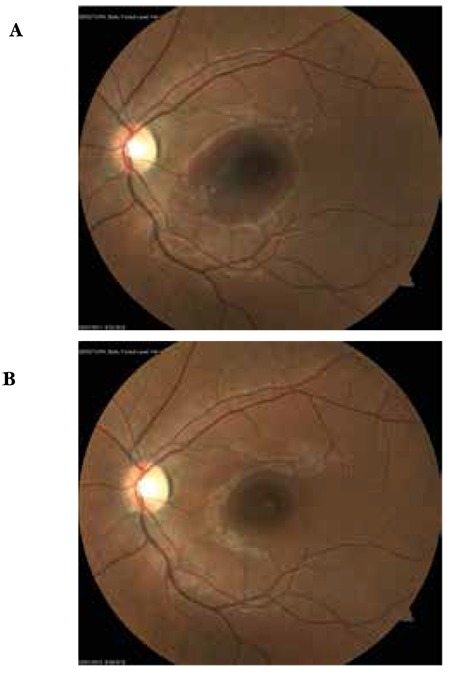
Case 3, color fundus photographs before (A) and after (B) intravitreal injection

**Figure 7:**
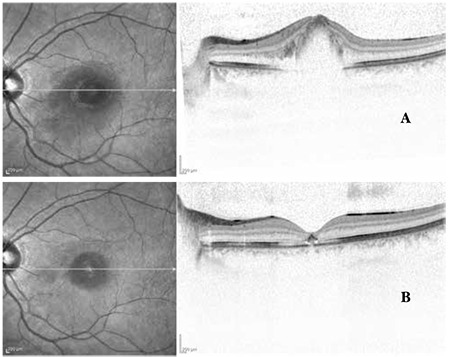
Case 3, optical coherence tomography images before (A) and after (B) intravitreal injection

**Figure 8:**
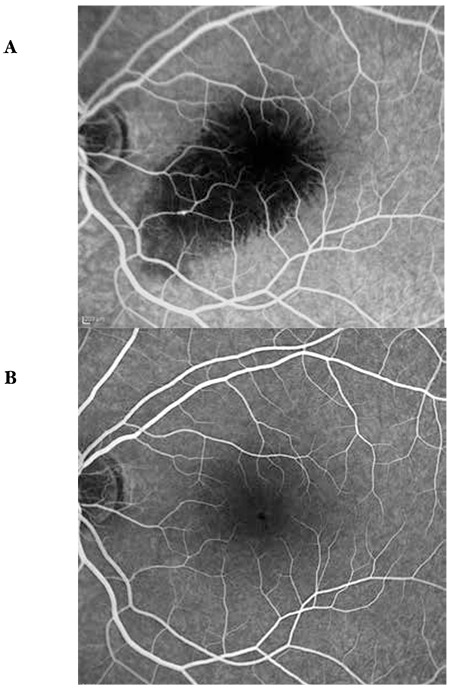
Case 3, fluorescein angiography images before (A) and after (B) intravitreal injection

**Figure 9:**
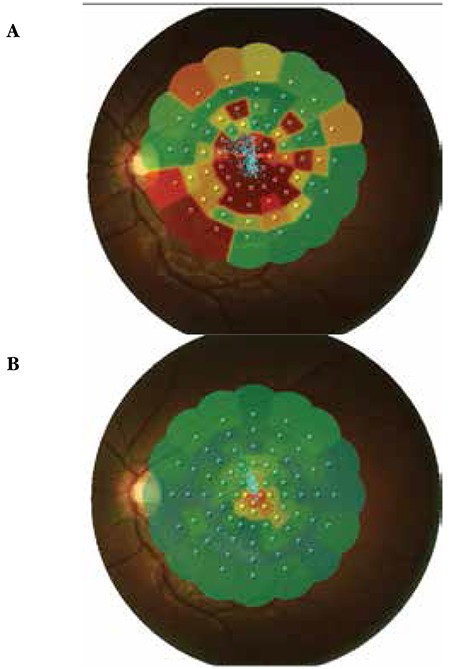
Case 3, microperimetry-1 images before (A) and after (B) intravitreal injection

A 24-year-old female patient presented to our clinic with complaints of vision loss in her left eye. She reported laser light shining into her left eye at an entertainment venue one week earlier, after which her vision problem started. On ophthalmologic examination, her BCVA was 10/10 in the right eye and 1/10 in the left eye. Anterior segment examination was normal and IOP was 11 mmHg in both eyes. Fundus examination of the right eye revealed subretinal hemorrhage in an area centered around the fovea covering approximately 3 disc diameters; fundus examination of the right eye was normal (Figure 6A). On OCT performed the same day, no pathologic findings were detected in the right eye, while intraretinal hemorrhage at the border of the outer plexiform layer and subretinal fluid were observed in the left eye. Increased reflectivity of the deeper retinal layers were noted (Figure 7A). Fluorescein angiography (FA) of the left eye revealed hypofluorescence due to blockage caused by hemorrhage, although the retinal vessels over the hemorrhage appeared healthy (Figure 8A). On MP1 analysis, fixation was central and stable and retinal sensitivity in the central 20 degrees was 17.0 dB in the right eye. In the left eye, fixation was extrafoveal and unstable, and retinal sensitivity was 7.8 dB (Figure 9A).

After informing the patient about her current condition, natural disease course, and the risks and success rates associated with the treatment, a total of 3 intravitreal anti-vascular endothelial growth factor (VEGF) injections were applied at 1-month intervals. Follow-up examinations were done at 1 day, 1 week, 3 months and 6 months after the injections. At 6 months her BCVA was 10/10 in the right eye and 3/10 in the left eye. Anterior segment examination was normal and IOP was 14 mmHg in both eyes. On fundus examination, we observed a reduction in the size of the foveal lesion (Figure 6B). The fovea appeared atrophic on OCT. The ELM was intact. A hyperreflective area was observed on the ELM at the foveal pit. IS/OS junction defect was noted (Figure 7B). FA was normal (Figure 8B). MP1 revealed the scotoma had regressed, while fixation was nearer to central and was relatively unstable. Retinal sensitivity was measured as 16.8 dB (Figure 9B).

## DISCUSSION

With the widespread use of lasers in various fields and the retina’s sensitivity to laser light, laser-induced ocular injuries are commonly encountered in ophthalmology practice. The American National Standards Institute defined four classes of laser device based on their potential risk.5 In this classification system, red-orange lasers with up to 1 mW of output power and longer wavelengths are in class II; green-blue lasers with up to 5 mW output power and shorter wavelengths are Class III. Exposure to class III and IV lasers can result in injury to the eyes and skin.^2,4,5^ Laser-induced ocular injuries may result from ablative, thermal or photochemical mechanisms depending on factors related to both the laser and the eye, including laser power, wavelength, spot size, exposure time, pupil diameter, proximity to the fovea, and amount of retinal and choroidal pigmentation.^2,4^ The main laser-related determining factor in retinal damage is the wavelength of the incident light.^2^ Visible and near-infrared incident light with wavelength between 380 and 1400 nm damages the retina. The damage increases in severity with longer exposure times. The blinking reflex and flinching in response to laser light limits laser exposure to between 0.15 and 0.25 seconds; these mechanisms serve as natural protection against laser-induced damage. Other important laser-related factors determining damage severity are pulse duration and energy level. High-energy and short pulses cause more damage to the retina.^2^ In our cases, we could not learn specific details regarding the wavelength, power and duration of the lasers our patients were exposed to; therefore, we do not know the laser class and duration of exposure that caused their retinal injuries. However, there are many reports in the literature of macular damage caused by class IIIA and higher lasers.^6,7,8,9,10^ The main retinal injuries reported related to class IIIA lasers are retinal pigment epithelium alterations; subretinal, intraretinal, subhyaloid and vitreous hemorrhages; epiretinal membrane; and full-thickness macular holes. The prefoveal hemorrhage observed in two of our cases and subfoveal hemorrhage in the other suggest that the lasers were Class IIIA.

The most important eye-related determining factor in laser-induced ocular damage is its localization on the retina. The resulting functional loss increases proportionately to the proximity of the damage to the fovea. With increasing distance from the fovea, the resulting scotomas are usually asymptomatic. Another factor related to the eye is pupil size. Because pupil dilation in dark environments allows more light to reach the retina, the resulting damage is more severe than that incurred in light environments. Furthermore, in individuals with more retinal and choroidal pigmentation, melanin absorbs more laser light, which leads to more severe injuries.^2,4^

Laser-induced hemorrhage may occur in different layers of the retina, and treatment options vary based on which layer is involved. In a report by Alsulaiman et al.^9^ of seven cases of intraocular hemorrhage associated with high-power lasers, five patients developed subhyaloid hemorrhage and two patients sub-ILM hemorrhage. Neodymium: yttrium-aluminum-garnet (Nd:YAG) hyaloidotomy was performed in all patients with subhyaloid hemorrhage; the procedure resulted in a rapid improvement in vision in three patients, but was unsuccessful in the other two. The two patients with sub-ILM hemorrhage were followed; during follow-up their hemorrhages spontaneously regressed and visual acuity improved. Similarly, two of our cases developed subhyaloid foveal hemorrhage, but unlike the other reports we achieved anatomic and functional success in these cases with surgery including 23-G PPV, posterior hyaloid dissection and ILM peeling.

The humanized monoclonal antibody bevacizumab (Avastin; Genentech /Roche, San Francisco, CA, USA) is used in the treatment of many retinal diseases, primarily choroidal neovascularization and age-related macular degeneration. Bevacizumab selectively inhibits VEGF, prevents abnormal vasculature formation and limits vascular permeability.^11,12^ Considering these effects of anti-VEGF therapy, we applied a total of three intravitreal anti-VEGF injections at one-month intervals in our patient with subretinal hemorrhage, despite the lack of clear guidance on this topic. An increase in visual acuity was achieved, atrophy at the fovea and damage at the IS/OS border resulted in permanent functional loss.

Another type of laser-related retinal injury is full-thickness macular holes. In a study by Alsulaiman et al.,9 laser-induced full-thickness macular hole was observed in four cases; anatomic and functional success was achieved in these patients with PPV, ILM peeling and silicone or gas tamponade injection.

## CONCLUSION

Outside the medical and military fields, the use of lasers ranging from 5 to 1200 mW has become very common in entertainment centers, presentations and meetings or as toys. Our natural protective mechanisms are insufficient for lasers of this power, and extremely severe, permanent functional losses may result. Restraining the uninformed use of high-power lasers by the general public through legislation and educating the public about the harmful effects of laser light are crucial to prevent laser-related permanent retinal injuries.

## Ethics

Informed Consent: In accordance with the principles of the Declaration of Helsinki, patients were informed about their current status and natural course, consent was obtained.

Peer-review: Externally peer-reviewed.
